# A Seven-Year Study of Carbapenem-Resistant *Klebsiella pneumoniae* Bloodstream Infections in a Tertiary Hospital in Greece: A Shift Toward Metallo-β-Lactamase and Dual Carbapenemase Strains

**DOI:** 10.3390/antibiotics15050491

**Published:** 2026-05-13

**Authors:** Eleni Mylona, Sofia Kostourou, Dimitroula Giankoula, Chrysoula Kolokotroni, Paraskevas Tsilikis, Nikolaos Koudoumnakis, Maria Papagianni, Dimitris Kounatidis, Natalia Vallianou, Efstathia Perivolioti, Vasileios Papastamopoulos

**Affiliations:** 1 Fifth Department of Internal Medicine and Infectious Diseases, Evaggelismos General Hospital, 10676 Athens, Greece; marianthi.p@gmail.com (M.P.); vasileios.papastamopoulos@gmail.com (V.P.); 2 Infection Control Committee, Evaggelismos General Hospital, 10676 Athens, Greece; kostsofia@gmail.com (S.K.); chrisakolokotroni@gmial.com (C.K.); 3 Center for Clinical Epidemiology and Outcomes Research (CLEO), 15451 Athens, Greece; dimitragiank@gmail.com; 4 Microbiology Department, Evaggelismos General Hospital, 10676 Athens, Greece; tsilikispar@gmail.com (P.T.); nikosdoudou@hotmail.com (N.K.); perivolioti@yahoo.gr (E.P.); 5 Diabetes Center, First Propaedeutic Department of Internal Medicine, Medical School, National and Kapodistrian University of Athens, Laiko General Hospital, 11527 Athens, Greece; dimitriskounatidis82@outlook.com; 6 First Department of Internal Medicine, Sismanogleio General Hospital, 15126 Athens, Greece; natalia.vallianou@gmail.com

**Keywords:** *Klebsiella pneumoniae*, carbapenem-resistant Enterobacterales (CRE), carbapenemases, KPC, metallo-β-lactamase (MBL), NDM, VIM, antimicrobial resistance, bloodstream infections, COVID-19, epidemiology, Greece

## Abstract

**Background/Objectives:** Carbapenem-resistant *Klebsiella pneumoniae* (CRKp) remains a critical driver of antimicrobial resistance (AMR) in hospital settings worldwide. **Methods:** This study examined trends in CRKp bloodstream infections over a seven-year period (2019–2025) in a tertiary care hospital in Greece, with particular attention given to resistance patterns and patient outcomes, including the impact of the COVID-19 pandemic. **Results:** A total of 671 non-duplicate CRKp isolates were analyzed and classified into three groups: KPC producers (67.4%), dual carbapenemase producers (dual CP) (17.4%), and single metallo-β-lactamase (MBL) producers (15.2%). Overall incidence showed a slight but non-significant increase over time. KPC-producing strains rose significantly until 2022 (*p* < 0.001), followed by a marked decline (*p* < 0.001). In contrast, dual CPs—mainly KPC combined with VIM or NDM—and single-MBL producers, particularly NDM, increased steadily, indicating a notable epidemiological shift. Resistance to aminoglycosides and tigecycline increased around 2021, followed by partial declines, whereas colistin resistance demonstrated a continuous upward trend throughout the study period. Despite phenotypic differences, overall mortality remained high, with no statistically significant differences between groups (*p* = 0.37), likely reflecting either the severity of patients’ clinical condition or inadequate empirical antibiotic therapy. **Conclusions:** This study highlights a dynamic evolution in CRKp epidemiology with decreasing KPC dominance and increasing prevalence of MBL- and dual CP strains. This transition, which became evident during and after the COVID-19 pandemic, underscores ongoing epidemiological adaptation and the urgent need for improved antimicrobial stewardship, rapid diagnostics, and broader access to effective therapies.

## 1. Introduction

Carbapenem-resistant *Klebsiella pneumoniae* (CRKp) has emerged as a major global public-health threat. Elevated to the top position on the World Health Organization (WHO) bacterial priority pathogen list in 2024 (previously ranked 5th in 2017), the prevalence of CRKp has increased significantly over the past decade, with particularly high incidence in certain geographic regions [1]. Infected patients often develop severe healthcare-associated infections, which are associated with prolonged hospital stays, increased healthcare costs, and substantially higher mortality due to limited therapeutic options [1,2].

The rapid spread of CRKp is driven by both organism-specific and contextual factors. *K. pneumoniae* readily colonizes the human gastrointestinal tract, where asymptomatic rectal carriage serves as a reservoir for horizontal transmission among patients and contributes to frequent nosocomial outbreaks [3]. The organism’s ability to persist in the environment and spread efficiently between individuals complicates outbreak control, even under strict contact precautions and rigorous cleaning protocols [4]. Resistance determinants are commonly carried on mobile genetic elements, particularly conjugative plasmids, which facilitate interstrain and interspecies transfer and often co-localize with genes conferring resistance to multiple other antibiotic classes, further restricting treatment options [5]. Beyond clinical and hospital settings, CRKp has been detected in animals, food products, and environmental niches, implicating the food chain and ecological reservoirs in transmission dynamics and highlighting the need for a One Health approach [6].

The most common mechanism of carbapenem resistance in *K. pneumoniae* is the production of carbapenemases, categorized into several types: class A *Klebsiella pneumoniae* carbapenemase (KPC), class B Verona integron-encoded β-lactamase (VIM), New Delhi metallo-β-lactamase (NDM), Imipenemase β-lactamase (IMP), and class D oxacillinase-48 (OXA-48) [7]. CRKp first emerged in Greece in 2002 and expanded until 2006, effectively replacing extended-spectrum β-lactamase (ESBL) strains [8,9,10]. The rapid spread of KPC-producing *K. pneumoniae* around 2007 subsequently established it as the dominant CRKp clone [11,12]. NDM emerged in 2011 [13], while OXA-48 was identified in 2012 [14]; however, OXA-48 did not spread widely in Greece, despite being the predominant mechanism in neighboring countries [15].

The combination of high transmissibility, plasmid-mediated multidrug resistance, limited novel therapeutics, and widespread colonization reservoirs underscores the urgent need for improved surveillance, rapid diagnostics, robust infection-prevention strategies, and antimicrobial stewardship. In Europe, data from the Italian AR-ISS surveillance system identified regional clusters of KPC-producing *K. pneumoniae*, prompting national infection-prevention guidelines and cohorting practices that reduced carbapenem-resistant Enterobacterales (CRE) incidence by more than 50% within two years [16]. At the University Hospital of Crete, continuous antimicrobial resistance (AMR) surveillance revealed increasing multidrug-resistant (MDR) *A. baumannii* and *K. pneumoniae* infections. Restriction of carbapenem use and real-time prescriber feedback subsequently led to reduction in carbapenem consumption without increasing 30-day mortality [17].

Surveillance becomes particularly critical during pandemics. During the coronavirus disease 2019 (COVID-19) pandemic, several studies reported increases in AMR infections [18,19,20]. In the U.S., the Centers for Disease Control and Prevention (CDC) reported a 70% increase in infections caused by “nightmare bacteria” between 2019 and 2023, largely driven by carbapenem-resistant Enterobacterales [21]. In Greece, increases in extensively drug-resistant (XDR) and pandrug-resistant (PDR) Gram-negative pathogens were also observed during the COVID-19 pandemic [22,23].

The present study aimed to evaluate the epidemiology and shifts in resistance phenotypes of CRKp, assess trends in resistance to last-resort antibiotics, and examine the impact on patient survival over a seven-year period, including the COVID-19 pandemic, in a tertiary care hospital in Greece that served as a COVID-19 referral center.

## 2. Results

### 2.1. Description of Clinical Isolates

Over the seven-year study period, 671 CRKp isolates were recovered from consecutive, non-duplicate blood cultures of 457,301 hospitalized patients, corresponding to 2,009,029 patient-days. The distribution of isolates by hospital sector was as follows: 50% from the medical sector, 33% from the ICU, and 13% from the surgical sector. Among all CRKp isolates, 67.4% were KPC producers, while 32.6% exhibited resistance to newer BL/BLI combinations, consistent with metallo-β-lactamase (MBL) production. Within the MBL group, 53.4% (17.4% of all CRKp isolates) produced dual carbapenemases, and 46.6% (15.2% of all CRKp isolates) carried a single MBL (NDM or VIM). No isolates producing IMP or OXA-48-like carbapenemases were identified.

**Figure 1:**
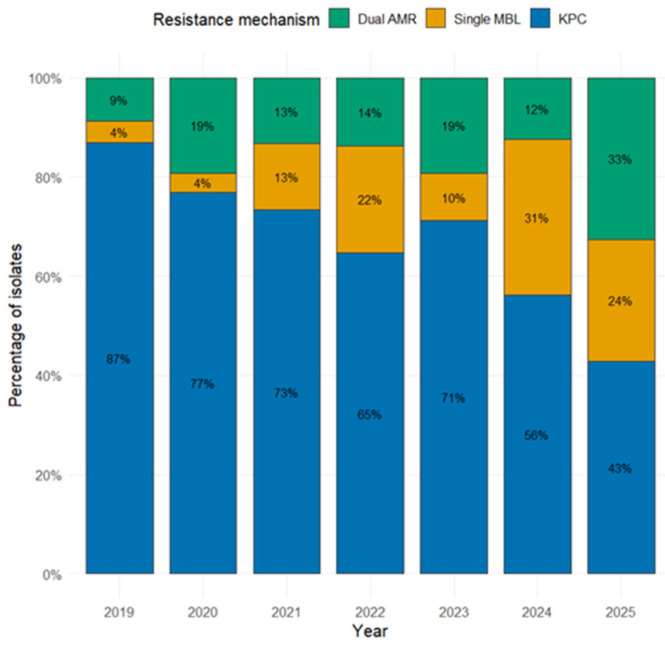
Annual distribution of resistant mechanisms.

The proportional representation of all CRKp phenotypes is summarized in Table 1. The annual distribution of resistant phenotypes, classified as KPC, dual CP, or single MBL-producing isolates, is presented in Figure 1.

### 2.2. Trend of Total CRKp in the Hospital and by Sector

**Figure 2:**
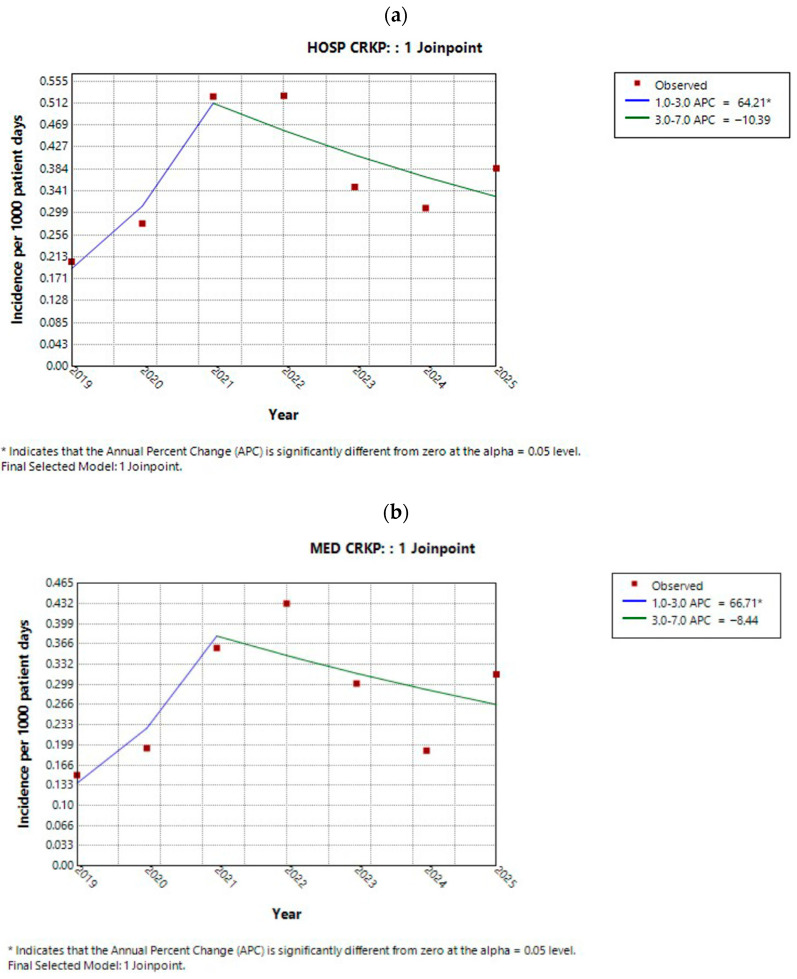

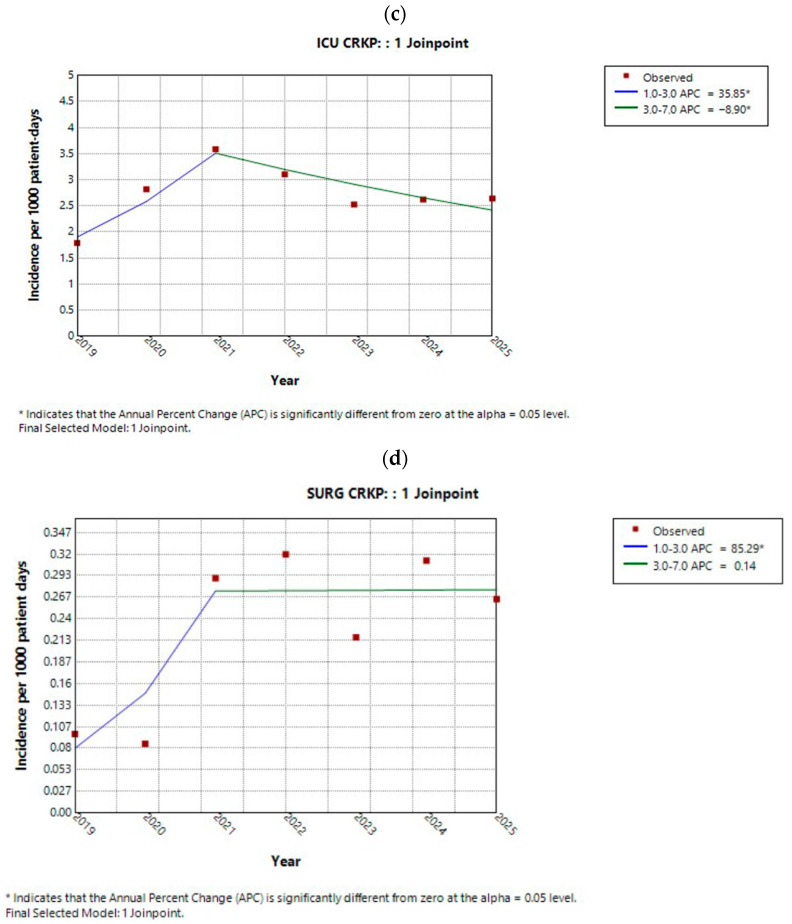
Trend of overall CRKp incidence in (**a**) the entire hospital, (**b**) the medical sector, (**c**) the ICU, and (**d**) the surgical sector during the study period.

Across the entire hospital, the annual trend of CRKp incidence followed the pattern illustrated in Figure 2a. Specifically, CRKp incidence increased annually from 2019 to 2021 (APC 64.2%, 95% CI 16.5 to 183.6%, *p* = 0.01), followed by a decline till 2025 (APC −38.9%, 95% CI −38.9 to 0.01%, *p* = 0.05). Sector-specific analysis (medical, ICU, and surgical) revealed distinct patterns. In both the medical sector (Figure 2b) and the ICU (Figure 2c), CRKp incidence exhibited a similar trend, with a transient peak around 2021 followed by a subsequent decrease. In contrast, in the surgical sector (Figure 2d), CRKp incidence increased sharply until 2021 and then remained relatively stable thereafter. The AAPCs for the hospital overall and for each sector are presented in Table 2. Owing to the observed “increase–decrease” pattern in APCs, the AAPCs for the overall study period were not statistically significant for the hospital, the medical sector, or the ICU. On the contrary, the surgical sector demonstrated a statistically significant upward trend over the study period.

### 2.3. Incidence Trends per CRKp Phenotype in the Hospital and by Sector

#### 2.3.1. Incidence Trends of KPC

**Figure 3:**
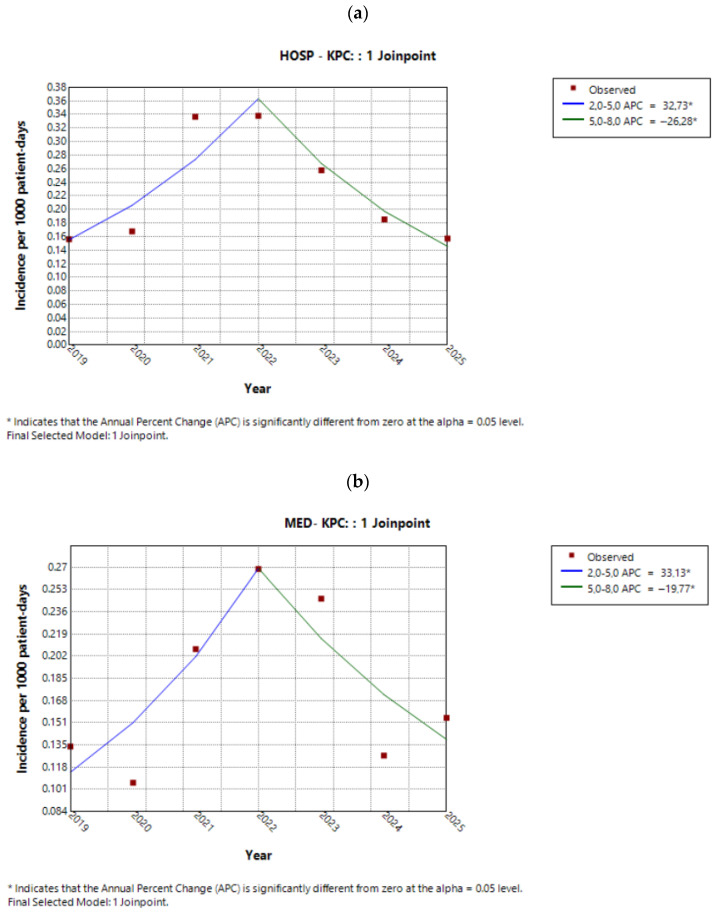

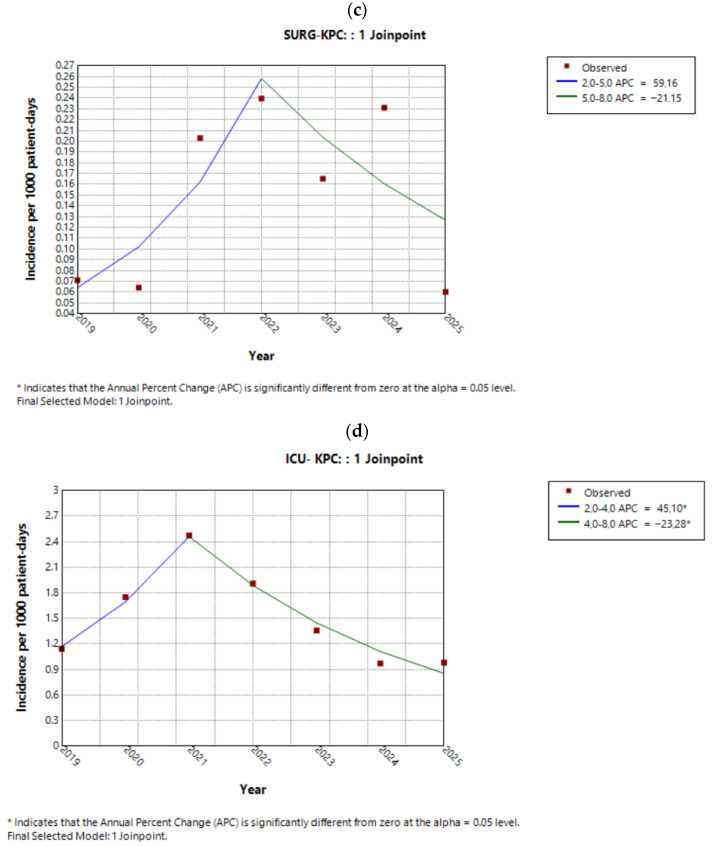
Trend of the KPC phenotype in (**a**) the entire hospital, (**b**) the medical sector, (**c**) the surgical sector, and (**d**) the ICU during the study period.

Across the hospital, the annual trend of the KPC phenotype followed the pattern illustrated in Figure 3a. Specifically, KPC incidence increased annually from 2019 to 2022 (APC 32.7%, 95% CI 15.1 to 68.0%, *p* < 0.001), followed by a significant decline till 2025 (APC −26.3%, 95% CI −42.2 to −14.46%, *p* < 0.001). Despite these significant changes within subperiods, the AAPC over the entire study period was not statistically significant (AAPC −1.07%, 95% CI −8.12 to 6.3%, *p* = 0.7).

Sector-specific analyses showed that KPC trends largely mirrored the overall hospital pattern. From 2019 to 2022, KPC incidence increased in both the medical sector (APC 33.1%, 95% CI 12.0 to 102.4%, *p* < 0.001; Figure 3b) and the surgical sector (APC 59.2%, 95% CI −2.1 to 392.4%, *p* = 0.054; Figure 3c), although the increase in the surgical sector was of borderline statistical significance. This was followed by a decline, which reached statistical significance only in the medical sector (APC −19.8%, 95% CI −54.0 to −5.1%, *p* = 0.007). In the ICU, an “increase–decrease” pattern was observed, with a significant rise up to 2021 (APC 45.1%, 95% CI 34.9 to 59.2%, *p* < 0.001), followed by a significant decline thereafter (APC −23.3%, 95% CI −27.3 to −20.4%, *p* < 0.001) (Figure 3d).

The AAPCs across all sectors are presented in Table 3. Although an “increase–decrease” pattern was evident in all sectors, only the ICU demonstrated a statistically significant overall downward trend (AAPC −5.1%, 95% CI −8.2 to −2.3%, *p* < 0.001).

#### 2.3.2. Incidence Trends of Dual Carbapenemase Producers

**Figure 4:**
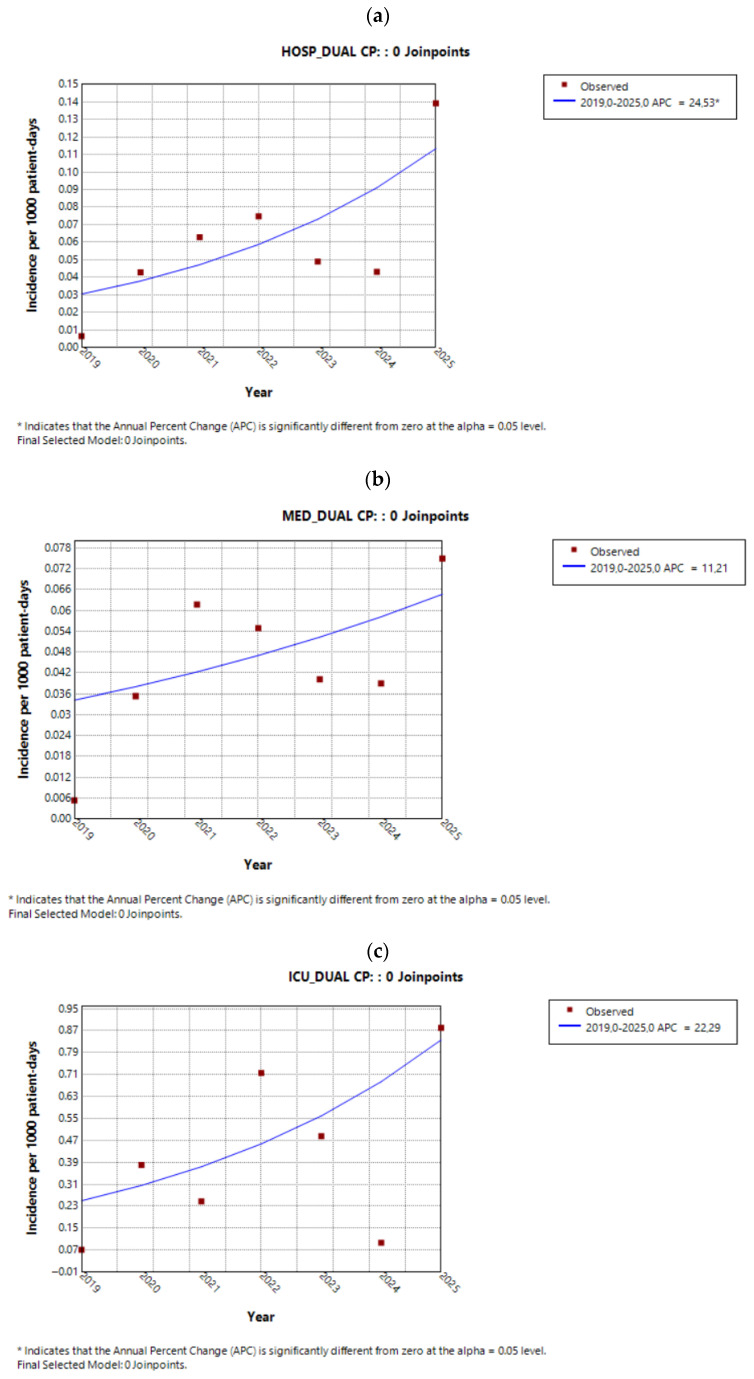

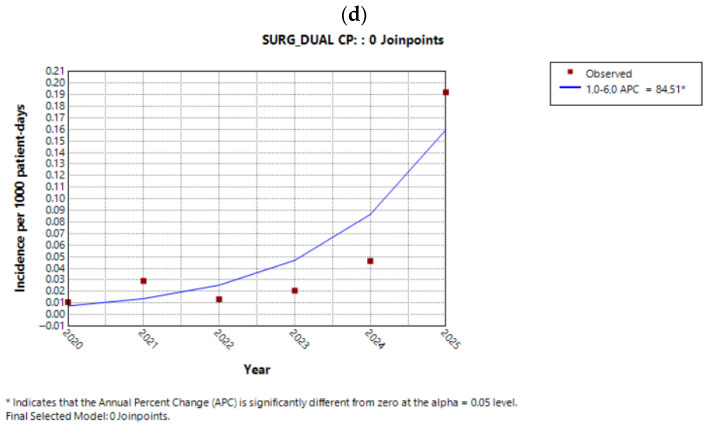
Trends of dual carbapenemase-producing (dual CP) isolates in (**a**) the entire hospital, (**b**) the medical sector, (**c**) the ICU, and (**d**) the surgical sector during the study period.

At the hospital level, isolates harboring dual carbapenemase resistance mechanisms exhibited a consistent and statistically significant increase over time, with equal APC and AAPC values (APC/AAPC 24.5%, 95% CI 6.0 to 57.6%, *p* = 0.005), as shown in Figure 4a. Sector-based analysis demonstrated a rising trend in dual carbapenemase-producing (dual CP) isolates across all sectors (Figure 4b,c); however, this increase reached statistical significance only in the surgical sector (Figure 4d). The AAPCs for each sector are presented in Table 4.

#### 2.3.3. Incidence Trends of Single-MBL Producers

**Figure 5:**
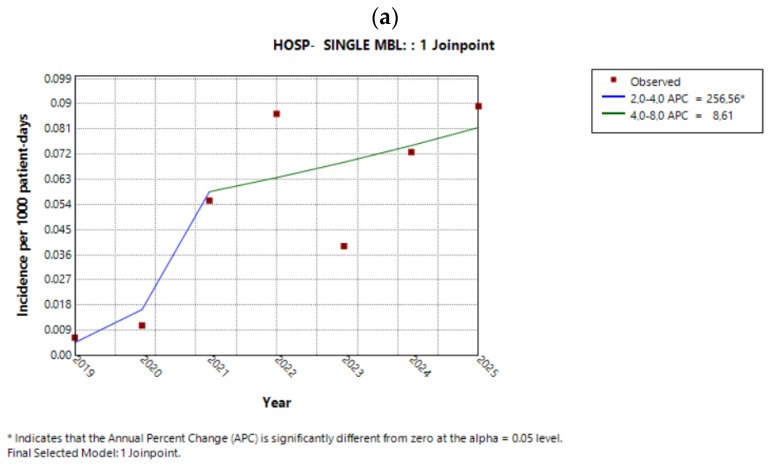

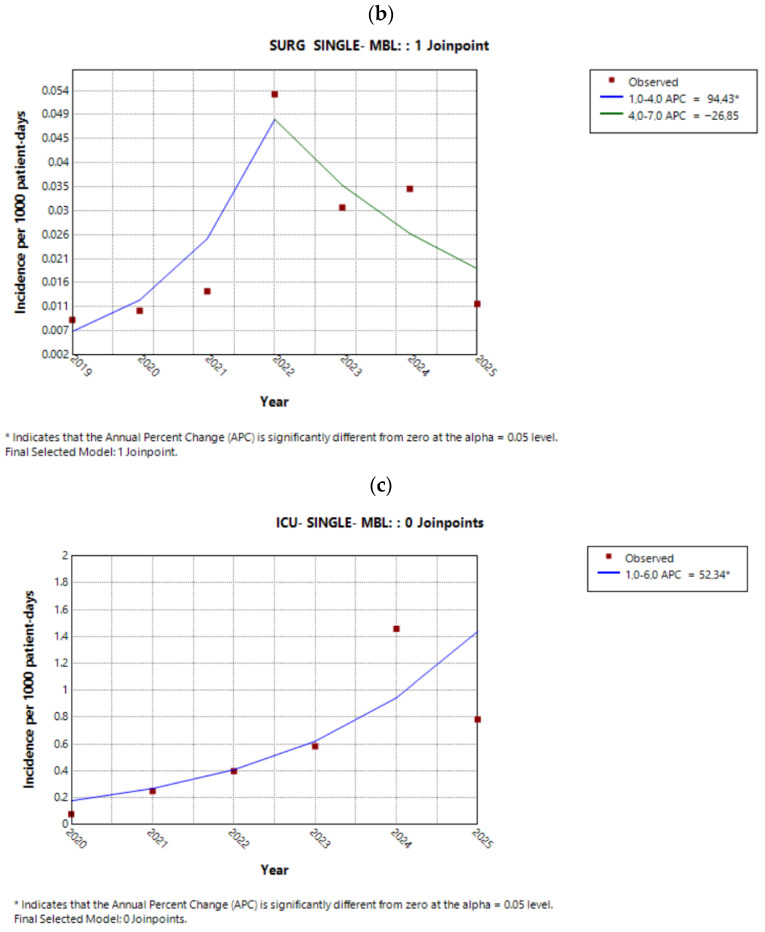
Trends of the single-MBL phenotype in (**a**) the entire hospital, (**b**) the surgical sector, and (**c**) the ICU during the study period.

At the hospital level, isolates exhibiting a single MBL resistance phenotype increased significantly until 2021 (APC 256.6%, 95% CI 75.4 to 1233.9%, *p* = 0.003). Thereafter, the upward trend persisted but was not statistically significant (APC 8.6%, 95% CI −21.2 to 31.3%, *p* = 0.48) (Figure 5a). Nevertheless, the average AAPC over the entire study period indicated a significant overall increase (AAPC 61.4%, 95% CI 33.5 to 149.9%, *p* < 0.001). Sector-based analysis demonstrated significant increases in single-MBL-producing isolates in both the surgical sector (Figure 5b) and the ICU (Figure 5c). The AAPCs for each sector are presented in Table 5.

#### 2.3.4. Incidence Trends of CRKp Resistance to Last-Resort Antibiotics

**Figure 6:**
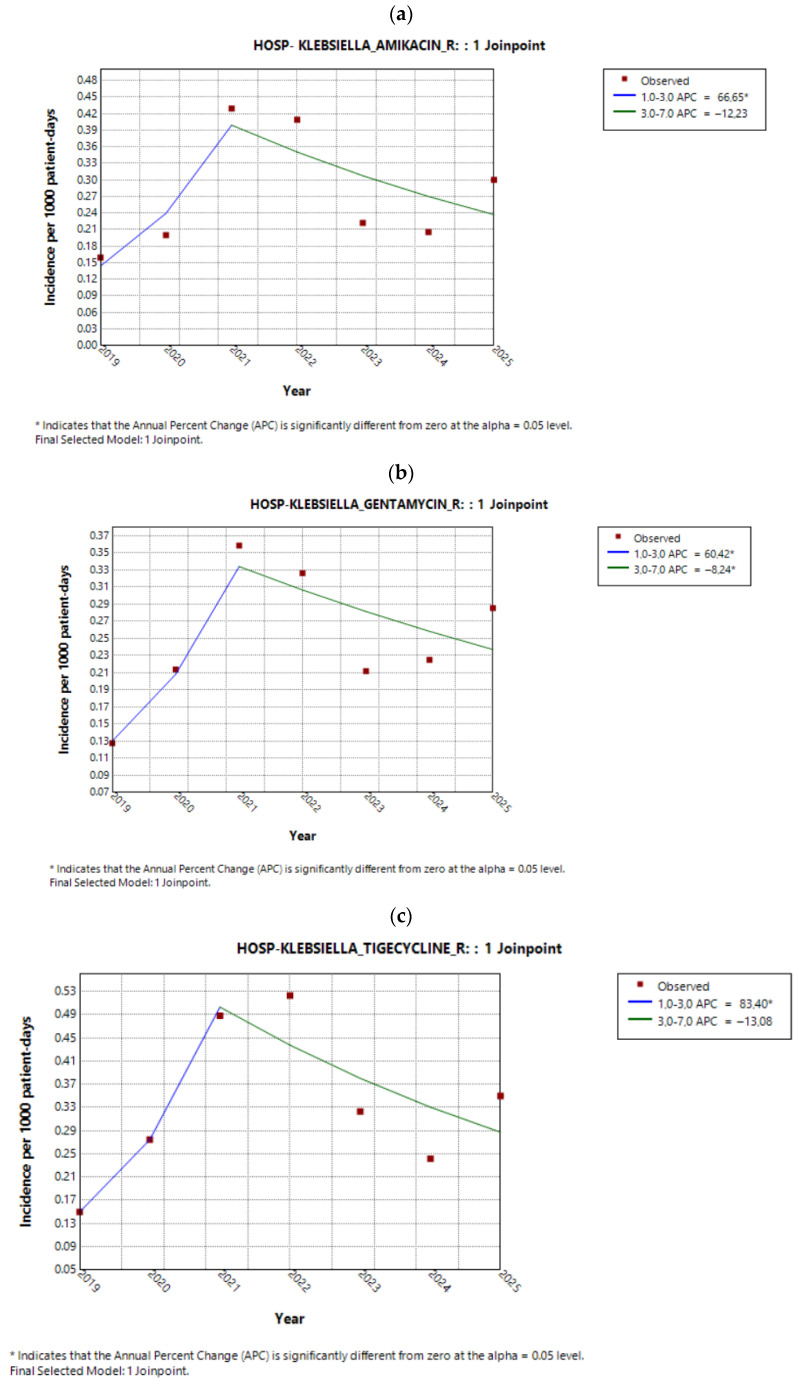

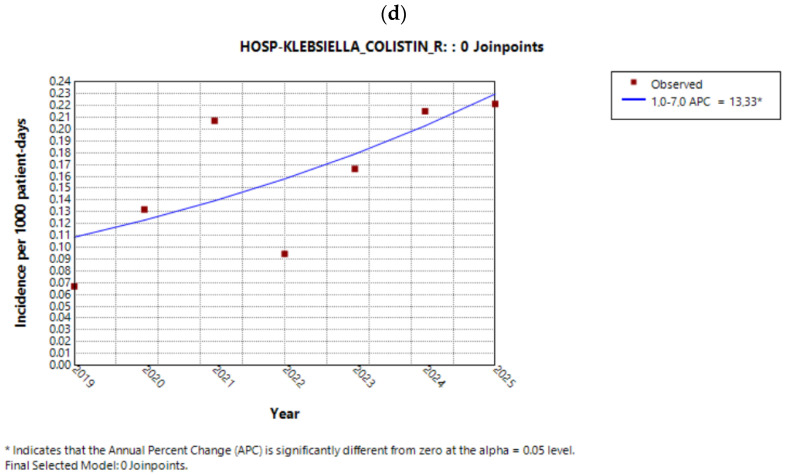
Annual trends in CRKp resistance to (**a**) amikacin, (**b**) gentamicin, (**c**) tigecycline, and (**d**) colistin during the study period.

The annual trends of CRKp resistance to amikacin, gentamicin, and tigecycline are presented in Figure 6a–c. All three exhibited a similar pattern, with a significant increase up to 2021, followed by a subsequent decline. This decrease was statistically significant but of borderline magnitude for amikacin and tigecycline. As a result, the overall AAPC across the study period was not statistically significant for these antibiotics (Table 5). In contrast, resistance to colistin demonstrated a continuous and sustained increase from 2019 to 2025 (Figure 6d, Table 6).

### 2.4. Patients’ Survival

Initial analyses compared mortality across resistance groups and hospital sectors. Given that dual carbapenemase and single MBL producers share similar resistance phenotypes in AST, mortality was first compared between KPC and non-KPC infections (the latter including both dual carbapenemase and single-MBL producers). Mortality was higher among non-KPC infections compared with KPC infections (51.3% vs. 43.2%), although this difference was of borderline statistical significance (χ^2^ = 3.66, *p* = 0.052). When analyzed across the three phenotypic categories, mortality was higher in infections caused by single-MBL-producing isolates (52.7%) compared with KPC infections (43.2%); however, this difference did not reach statistical significance (χ^2^ = 4.15, *p* = 0.126). In contrast, mortality differed significantly across hospital sectors (χ^2^ = 13.44, *p* = 0.0038), with the highest mortality observed in the ICU and medical wards, and the lowest in the surgical wards (Table 7).

Survival analysis was subsequently performed using the Kaplan–Meier method to estimate survival following *K. pneumoniae* bloodstream infection. Survival time was defined as the interval from the first positive blood culture to death or the end of follow-up. Patients without documented death were handled according to predefined censoring rules. In cases where follow-up data were unavailable (e.g., due to transfer to another hospital or incomplete electronic records), patients were conservatively classified as deceased to avoid underestimation of mortality.

**Figure 7:**
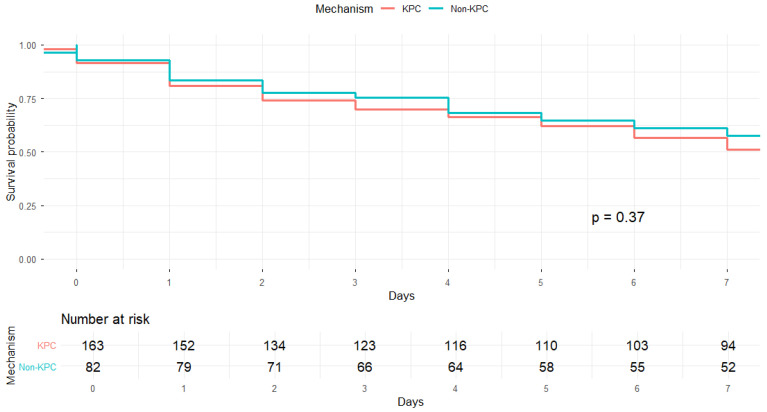
Kaplan–Meier curve of 7-day cumulative mortality according to carbapenemase profile (KPC vs. non-KPC infections).

**Figure 8:**
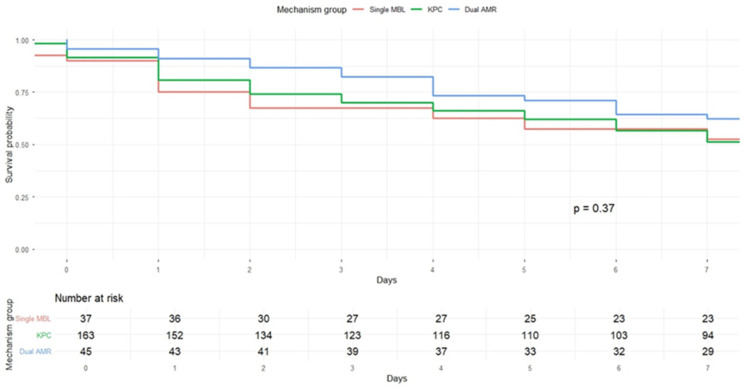
Kaplan–Meier curve of 7-day cumulative mortality according to CRKp phenotype (KPC, dual carbapenemase-producing, and single-MBL infections).

Two Kaplan–Meier analyses were conducted. In the first, patients were stratified into KPC versus non-KPC groups. No statistically significant difference in 7-day survival was observed between these groups (log-rank χ^2^ = 0.8, *p* = 0.37) (Figure 7). In the second analysis, patients were stratified into three resistance mechanism groups: KPC, single-MBL, and dual CPs. The median survival time was 8 days (95% CI 6–11) for KPC infections, 8 days (95% CI 4–19) for single-MBL infections, and 18 days (95% CI 7–25) for dual CP infections. Despite the numerically longer survival observed in the dual-mechanism group, the substantial overlap in confidence intervals indicated no statistically significant difference in survival among the groups (*p* = 0.37) (Figure 8).

To further characterize survival dynamics, the number of patients at risk over time was examined. A rapid decline in the number at risk was observed within the first 10 days across all groups, indicating substantial early mortality. Among patients with KPC infections, the number at risk decreased from 163 at baseline to 72 at day 10 and 30 at day 30. Similar patterns were observed in the dual carbapenemase group (45 to 27 to 12) and the single-MBL group (37 to 19 to 6) (Table 8), underscoring the high early mortality across all phenotypic categories.

## 3. Discussion

The present study aimed to comprehensively evaluate temporal trends in the incidence of CRKp, the distribution of its phenotypic categories (KPC, dual carbapenemase, and single-MBL producers) across the overall hospital setting and individual sectors, patterns of resistance to last-line antimicrobial agents, and their impact on patient survival.

The principal findings can be summarized as follows. First, at the hospital-wide level, as well as within the medical sector and ICU, a modest and non-statistically significant increase in CRKp incidence was observed over the seven-year period. This trend was characterized by a pronounced surge peaking in 2021–2022, followed by a decline approaching baseline levels observed in 2019. In contrast, in the surgical sector, the increased incidence observed in 2021 persisted without substantial decline through 2025. Second, the peak in CRKp incidence during 2021–2022 coincided with a corresponding rise in KPC-producing isolates, followed by a marked reduction. This reduction returned KPC levels to those comparable to 2019 in the medical and surgical sectors and to even lower levels in the overall hospital and ICU. This shift was accompanied by an apparent epidemiological replacement of KPC producers by isolates harboring dual carbapenemases (primarily KPC combined with VIM or NDM) and by single-MBL phenotypes, predominantly NDM. Third, resistance to amikacin, gentamicin, and tigecycline followed a similar temporal pattern, with increases around 2021 and partial declines thereafter, although rates did not return to pre-2020 levels. In contrast, resistance to colistin increased steadily throughout the study period. Finally, mortality remained high and tended to be greater among infections caused by MBL-harboring strains compared with KPC producers; however, no statistically significant differences were observed in survival analyses.

Following the emergence of VIM carbapenemase in Greece in 2002 [8], it predominated in Greek hospitals, including our own, until 2007 [10], when it was progressively replaced by KPC [11,12]. Since then, KPC has remained endemic, accounting for approximately 66.5% of cases, followed by NDM and VIM, while OXA-48-like carbapenemases remain rare [15,24]. Surveillance data from the European Centre for Disease Prevention and Control (ECDC) demonstrated an increasing trend in CRKp incidence in Greece from 2019, reaching 73.7% in 2021 [25]. Our findings are consistent with these data, showing a peak in CRKp incidence in 2021–2022, largely driven by KPC-producing isolates [24]. Notably, a sharp increase in single-MBL-producing isolates, predominantly NDM, was also observed during the same period.

These increases are likely attributable to the COVID-19 pandemic. During this period (March 2021 to December 2022), our hospital functioned as a mixed facility managing both COVID-19-positive (ICU and part of the medical wards) and non-COVID patients (remaining medical wards and surgical ward). Prolonged hospitalizations, increased ICU admissions, and the widespread use of broad-spectrum antibiotics due to concerns about secondary bacterial infections have been widely associated with increased antimicrobial resistance (AMR) [26,27,28]. In addition, disruptions in infection prevention and control (IPC) practices driven by increased workload—such as reduced adherence to hand hygiene, suboptimal equipment decontamination, inadequate cohorting, and inconsistent use of personal protective equipment—likely facilitated transmission [29]. The continuous transfer of patients between COVID-19 wards, ICUs, and non-COVID units may have further amplified the spread of resistant organisms. This is supported by our observation that KPC incidence peaked earlier in the ICU (2021) and later in the medical and surgical wards (2022), shaping the overall hospital trend.

After 2022, improved antimicrobial stewardship and stricter IPC measures were associated with a decline in CRKp incidence, although rates remained higher than in 2019. Importantly, KPC ceased to predominate and was progressively replaced by both dual carbapenemase and single-MBL mechanisms. Dual carbapenemase-producing strains have been increasingly reported in Greece since the first description of KPC + VIM in 2009 [30], followed by additional combinations such as NDM + VIM (2016) [31], NDM + OXA-48-like (2019) [32], and KPC + NDM (2022) [33]. Although typically reported at low prevalence (2.5–7.7%) [12,24,33,34,35,36,37], higher rates, up to 33%, have been observed during outbreaks [38]. Similar patterns have also been described in other regions [39,40,41,42,43,44], where the NDM + OXA-48-like combination often predominates [39,44].

To our knowledge, this is the first study demonstrating a progressive replacement of long-standing endemic KPC by single-MBL (primarily NDM) and dual carbapenemase-producing strains (mainly KPC + VIM and, to a lesser extent, KPC + NDM). At this point, it is worth noting that the co-presence of *bla*KPC-2 and *bla*VIM-1 has been reported in highly drug-resistant ST39 *K. pneumoniae* isolates from 2018 and 2019 [45]. Furthermore, a surveillance study by Tryfinopoulou et al. demonstrated that among 310 CRKp isolates collected from 15 Greek hospitals, all isolates carrying multiple carbapenemase genes, including *bla*KPC-2 with either *bla*VIM-1 or *bla*NDM, belonged to the ST39 lineage. This clone was shown to spread rapidly both within and between hospitals and has therefore been characterized as a high-risk clone [35]. Very recently, Eleftherakis et al. studied the molecular epidemiology of *Klebsiella pneumoniae* in bloodstream infections in Greece and reported that, among 100 molecularly typed isolates, strains co-harboring KPC and NDM (13%) or KPC and VIM (3%) belonged to either ST39 or ST512 lineage [46]. In the present study, molecular typing was not performed, and thus assignment of isolates to specific clones was not feasible. Consequently, it remains unclear whether the dual carbapenemase-producing isolates observed here belong to the ST39 or ST512 lineages, which could potentially explain their progressive dissemination in our hospital from 2022 onwards, or whether they represent the emergence of a novel clone. Nevertheless, irrespective of their clonal background, MBL-harboring strains (either single or dual) exhibited a highly drug-resistant phenotype and were associated with infections that are increasingly difficult to treat.

From a therapeutic perspective, KPC-producing isolates are generally managed with β-lactam antibiotics combined with newer β-lactamase inhibitors, whereas MBL-harboring isolates require more complex regimens, such as ceftazidime/avibactam plus aztreonam or aztreonam/avibactam [47]. In our hospital, until 2018, last-line agents, including aminoglycosides, colistin, tigecycline, and fosfomycin, constituted the only available treatment options for CRKp infections. Ceftazidime/avibactam was introduced into routine clinical use in 2019 under strict restriction policies to preserve its efficacy. It was administered only in cases of confirmed infection caused by KPC-producing isolates susceptible to ceftazidime/avibactam in critically ill patients having no other choices in AST such as colistin or aminoglycoside, or empirically in patients with septic shock known to be colonized with KPC [48,49]. The aforementioned practice resulted in the systematic use of colistin as part of both empirical and targeted therapy, which likely contributed to the progressive emergence of resistance to this agent over the seven-year study period.

Aztreonam availability in Greece has been inconsistent, with only intermittent access, while aztreonam/avibactam is not routinely available and can be obtained only through special request procedures. As a result, last-resort antibiotics continued to be widely used throughout the study period, both empirically and as targeted therapy, reflecting the limited availability of effective treatment options, particularly against MBL-producing organisms. Despite these therapeutic constraints, mortality was only marginally higher among infections caused by MBL-harboring strains compared with KPC-producing isolates, and Kaplan–Meier analysis did not demonstrate a statistically significant difference between groups. This finding, consistent with previous studies [45,47], together with the observed early mortality in our cohort, may reflect either suboptimal initial antimicrobial therapy across all CRKp phenotypes or the poor baseline clinical status of patients with severe underlying disease and multiple comorbidities.

The changing epidemiology of CRKp, characterized by the progressive replacement of KPC by MBL-harboring strains (predominantly dual CPs) suggests ongoing genetic exchange and selective antimicrobial pressure driving this evolution. Of particular concern is the potential horizontal transfer of MDR plasmids to other Enterobacterales, such as *E. coli*, as well as the dissemination of resistance genes into the community, similar to what has been previously observed with ESBLs. Moreover, evidence indicates that certain *K. pneumoniae* strains, including hospital-associated pathogens, can persist and proliferate across diverse ecological niches, such as the gastrointestinal tract of animals and environmental reservoirs like soil [50,51]. These environments facilitate genetic exchange with other bacterial species. Taken together, these characteristics highlight *K. pneumoniae* as a critical target for sentinel surveillance, particularly for the early detection of emerging antimicrobial resistance genes within Gram-negative pathogens [52].

Collectively, these findings underscore the urgent need to strengthen antimicrobial stewardship and, above all, to reinforce infection prevention and control measures [53]. In the context of rotating hospital admissions, limited isolation capacity, and the endemic presence of MDR pathogens, strict adherence to hand hygiene is of paramount importance. This should be complemented by consistent implementation of contact precautions, potentially applied universally, as if all patients were colonized with MDR organisms. However, such an approach may impose additional strain on an already understaffed healthcare system and further complicate routine clinical practice.

The main strength of the present study lies in its extended observation period of seven years, including three years following the official end of the COVID-19 pandemic, providing a comprehensive view of carbapenemase dynamics and epidemiological shifts. Nevertheless, several limitations should be acknowledged. The lack of detailed clinical data beyond survival outcomes limits the ability to characterize patient comorbidities and risk factors. Additionally, the absence of molecular typing precludes identification of circulating *K. pneumoniae* clones. Finally, the single-center design may limit generalizability; however, data from a large tertiary-care hospital are likely to reflect broader national trends, as supported by WHONET Greece surveillance data.

## 4. Materials and Methods

This retrospective surveillance study, aimed to investigate the incidence of CRKp, was conducted at Evaggelismos General Hospital, a 946-bed tertiary care center in Greece, over a seven-year period from January 2019 to December 2025. *Klebsiella pneumoniae* strains isolated from consecutive positive blood cultures of hospitalized patients, obtained for diagnostic purposes and representing true infections rather than colonization, were included. For each patient, only the first positive isolate was considered. Relevant clinical data and patient outcomes were collected from medical records.

### 4.1. Microbiological Methods

Clinical isolates were identified using conventional microbiological techniques, including subculturing on agar-based media, followed by biochemical identification using the Vitek 2 Compact System (bioMérieux, Marcy l’Etoile, France). Antibiotic susceptibility testing (AST) was performed using the minimum inhibitory concentration (MIC) method via the Vitek 2 system. In particular, *K. pneumoniae* isolates were tested against the following antibiotics: amoxicillin/clavulanic acid, ampicillin/sulbactam, ticarcillin, piperacillin, piperacillin/tazobactam, cefotaxime, ceftazidime, ceftriaxone, cefepime, aztreonam, ertapenem, meropenem, amikacin, gentamicin, tobramycin, ciprofloxacin, levofloxacin, moxifloxacin, minocycline, tetracycline, tigecycline, colistin, trimethoprim-sulfamethoxazole, ceftolozane/tazobactam, ceftazidime/avibactam, and fosfomycin. Colistin MICs were determined using the reference broth microdilution method (UMIC^®^ test strips, Bruker, Billerica, MA, USA), as recommended. Interpretation of antibiotic susceptibility followed EUCAST Clinical Breakpoint Tables, version 14.0 (effective from 1 January 2024), with isolates categorized as susceptible (including susceptible with increased exposure) or resistant [54]. As far as sensitivity to tigecycline is concerned, we followed the guidance document of tigecycline dosing of EUCAST (July 2022) according to which *K. pneumoniae* strains with MIC ≤ 1 mg/dL is anticipated to respond to treatment with high dose of tigecycline [55].

### 4.2. Carbapenemase Detection and Phenotype Classification

Carbapenemase production was assessed using an immunochromatographic assay (NG-TEST CARBA5, Biotech, Guipry, France), which detects and differentiates the five most prevalent carbapenemases: KPC, NDM, IMP, VIM, and OXA-48-like, either individually or in combination. Early identification of the resistance mechanism allowed targeted use of newer, restricted antimicrobials as part of the hospital’s antimicrobial stewardship program. Based on carbapenemase detection and AST profiles, isolates were classified into three phenotypic groups: (i) KPC strains, which are characterized by susceptibility to newer β-lactam/β-lactamase inhibitor (BL/BLI) combinations, including ceftazidime/avibactam, imipenem/relebactam, and meropenem/vaborbactam; (ii) single MBL strains (VIM or NDM), which exhibit resistance to the newer BL/BLI combinations; (iii) dual carbapenemase-producing strains (predominantly KPC + NDM, KPC + VIM, OXA-48-like + NDM and NDM + VIM), with resistance to the newer BL/BLIs combinations.

### 4.3. Data Analysis

The annual incidence of KPC, single MBL, and dual CP isolates was analyzed hospital-wide and by sector (medical, surgical, and intensive care unit [ICU]). Among all CRKp isolates, the annual incidence of resistance to aminoglycosides, tigecycline, or colistin, and the combined resistance to all three (PDR strains), was evaluated. Kaplan–Meier survival analysis was used to compare patient survival across the different phenotype groups. Hospital sectors were defined as follows: (i) medical sector: Internal Medicine, Cardiology, Nephrology and Kidney Transplant Unit, Neurology, Hematology–Oncology, Hematopoietic Stem Cell Transplant Unit, and the COVID-19 ward (operating March 2020–December 2022); (ii) surgical sector: General Surgery, Orthopedics, Neurosurgery, Urology, Maxillofacial Surgery, Otorhinolaryngology, Cardiothoracic Surgery, and Vascular Surgery; (iii) ICU: three adult units. Incidence rates were calculated per 1000 patient-days.

### 4.4. Ethical Considerations

All data were collected and processed in accordance with institutional and national ethical standards and the Declaration of Helsinki (1975, revised 2013). The study protocol was approved by the Ethics Committee of Evaggelismos General Hospital (protocol number 23339/18-07-2025).

## 5. Statistical Analysis

Trends in CRKp and CRKp phenotypes were analyzed using Joinpoint software, version 4.9.1.0 (National Cancer Institute, Bethesda, MD, USA) [56]. This regression-based method identifies year(s) in which significant changes in trend occur, calculates the annual percentage change (APC) for each trend segment along with the corresponding 95% confidence interval (CI), and estimates the average annual percentage change (AAPC) over the entire study period. The APC is tested against the null hypothesis of no change (0%). When no trend changes (i.e., no joinpoints) are present, APC equals AAPC; otherwise, the study period is segmented at statistically significant joinpoints, each indicating a statistically significant increase or decrease in the trend. In the diagrams, the red squares represent the observed rates based on the hospital’s annual data, while the line is generated from the modeled crude rates produced by the statistical software.

Mortality analyses were conducted using R statistical software via RStudio (version 2026.01.0 Build 392, Posit Software, PBC, Boston, MA, USA, “Apple Blossom” release) running on Windows, with Quarto version 1.8.25. Comparisons between categorical variables were performed using the Pearson χ^2^ (chi-square) test. All statistical tests were two-sided, and a *p*-value < 0.05 was considered statistically significant.

## 6. Conclusions

In conclusion, this surveillance study highlights a dynamic evolution in CRKp epidemiology with decreasing KPC dominance and increasing prevalence of MBL- and dual carbapenemase-producing strains that are increasingly resistant to last-resort antibiotics. This transition, which became evident during and after the COVID-19 pandemic, emphasizes ongoing epidemiological adaptation which limits therapeutic options as newer antibiotics, many of which remain inaccessible or unavailable, often represent the only effective treatments, potentially contributing to increased patient mortality. These findings highlight the urgent need for coordinated national and broader European strategies to combat AMR, including strengthened surveillance systems, improved access to effective antimicrobials, robust antimicrobial stewardship, access to rapid diagnostics and reinforced infection prevention and control measures.

## Figures and Tables

**Table 1:** Proportional distribution of CRKp phenotypes identified during the study period.

CRKp Phenotypes	Number of Isolates
KPC	452 (67.4)
NDM	92 (13.7)
KPC + VIM	78 (11.6)
KPC + NDM	22 (3.3)
NDM + OXA-48-like	15 (2.2)
VIM	10 (1.5)
NDM + VIM	2 (0.3)

Values in parentheses represent percentages. CRKp, carbapenem-resistant *Klebsiella pneumoniae*.

**Table 2:** Incidence trends of the overall CRKp, over the 7–year study period, estimated for the hospital and by sector. Time trends indicated by AAPCs, 95% CIs and *p*-values.

Domain	AAPC	Lower CI	Upper CI	*p*-Value
Hospital	9.6	–5.2	28.8	0.15
Medical	11.8	−3.5	33.5	0.12
ICU	4.1	−1.1	9.84	0.12
Surgical	22.9 *	11	38.7	<0.001

* Indicates that the AAPC is significantly different from zero at the alpha = 0.05 level.

**Table 3:** Incidence trends of KPC, over the 7–year study period, estimated by hospital sector. Time trends indicated by average annual percent changes (AAPCs), 95% confidence intervals (CIs) and *p*-values.

Sector	AAPC	Lower CI	Upper CI	*p*-Value
Medical	3.35	−8.3	17.5	0.57
Surgical	12.0	−16.1	59.2	0.35
ICU	−5.1 *	−8.2	−2.3	<0.001

* Indicates that the AAPC is significantly different from zero at the alpha = 0.05 level.

**Table 4:** Incidence trends of dual carbapenemase producers, over the 7–year study period, estimated by hospital sector, expressed as AAPCs with 95% CIs and *p*-values.

Sector	AAPC	Lower CI	Upper CI	*p* Value
Medical	11.2	−11.9	47.9	0.25
Surgical	84.5 *	26.1	369.1	0.001
ICU	22.3	−14.6	81.1	0.17

* Indicates that the AAPC is significantly different from zero at the alpha = 0.05 level.

**Table 5:** Incidence trends of single-MBL producers, over the 7–year study period, estimated by hospital sector, ex-pressed as AAPCs with 95% CIs and *p*-values.

Sector	AAPC	Lower CI	Upper CI	*p*-Value
Medical	16.3	−17.5	79.5	0.29
Surgical	19.2	−14.5	87.9	0.24
ICU	52.3 *	17.8	116.1	<0.001

* Indicates that the AAPC is significantly different from zero at the alpha = 0.05 level.

**Table 6:** Incidence trends of CRKp resistance to amikacin, gentamicin, tigecycline, and colistin over the 7–year study period, expressed as AAPCs with 95% CIs and *p*-values.

Antibiotic	AAPC	Lower CI	Upper CI	*p*-Value
Amikacin	8.7	−8.0	−30.5	0.27
Gentamycin	10.5 *	2.7	19.3	0.005
Tigecycline	11.5	−8.9	39.8	0.20
Colistin	13.3 *	1.3	30.5	0.03

* Indicates that the AAPC is significantly different from zero at the alpha = 0.05 level.

**Table 7:** Resistance mechanisms and admitting ward in relation to 7-day mortality.

Comparison	Group	Alive	Dead	Mortality	*p*-Value
MBL-harbouring	KPC	243	185	43.2%	
	Non-KPC	116	122	51.3%	0.05
CRKp phenotype	KPC	243	185	43.2%	
	Dual Carbapenemase	63	63	50.0%	
	Single-MBL	53	59	52.7%	0.12
Sector	Medical	178	172	49.1%	
	Surgical	82	40	32.8%	
	ICU	120	126	51.2%	0.003

**Table 8:** Number of patients at risk to die over time.

Group (Patients Survived till That Day)	Day 0	Day 5	Day 10	Day 15	Day 20	Day 25	Day 30
KPC	163	110	72	62	54	41	30
Dual	45	33	27	25	21	15	12
Single MBL	37	25	19	15	12	7	6

## Data Availability

The original contributions presented in this study are included in the article. Further inquiries can be directed to the corresponding author.
